# Model templates: transdisciplinary application and entanglement

**DOI:** 10.1007/s11229-023-04178-3

**Published:** 2023-06-02

**Authors:** Tarja Knuuttila, Andrea Loettgers

**Affiliations:** Department of Philosophy, University of Vienna, Universitätsstraße 7, A-1010, Vienna, Australia

**Keywords:** Models, Template transfer, Model templates, Interdisciplinarity, Transdisciplinarity, Network science, Statistical mechanics

## Abstract

The omnipresence of the same basic equations, function forms, algorithms, and quantitative methods is one of the most spectacular characteristics of contemporary modeling practice. Recently, the emergence of the discussion of templates and template transfer has addressed this striking cross-disciplinary reach of certain mathematical forms and computational algorithms. In this paper, we develop a notion of a model template, consisting of its mathematical structure, ontology, prototypical properties and behaviors, focal conceptualizations, and the paradigmatic questions it addresses. We apply this notion to three widely disseminated and powerful model templates: the Sherrington-Kirkpatrick model of spin glasses, scale-free networks, and the Kuramoto model of synchronization. We argue that what appears to be an interdisciplinary model transfer between different domains turns out, from a broader perspective, to be the application of transdisciplinary model templates across a multitude of domains. We also point out a further feature of template-based modeling that so far has not been discussed: template entanglement. Such entanglement enhances and makes manifest the conceptual side of model templates.

## Introduction

Much philosophical attention has been paid to the fact that different models may be used to represent the same natural or social systems. That a seemingly identical model may be applied to different domains, often studied by different disciplines, has gotten less attention. Yet such transdisciplinary application of models is pervasive in contemporary model-based theoretical practice. Several models that have also featured prominently in the philosophy of science discussions, such as the Lotka-Volterra model, the Ising model, and scale-free networks, can be found across mathematically and computationally oriented scientific domains, from the natural to the social sciences. While philosophers have certainly noted this dissemination of certain mathematical forms, methods, and the models in which they are embedded, they have mostly left it at that. One problem has been that of finding suitable analytic tools for addressing the cross-disciplinary reach of mathematical forms and computational algorithms. The recent discussion of templates and template transfer has made such analytic tools available.

The discussion of templates originates from Paul Humphreys’ notions of a theoretical template and a computational template (Humphreys, 2002, 2004). Subsequently, several case studies used these notions to examine interdisciplinary transfers of the Lotka-Volterra model (Knuuttila & Loettgers 2011, 2017; Houkes & Zwart, 2019), the Ising model (Knuuttila & Loettgers, 2014, 2016b), the virial theorem and the ideal gas law (Price, 2019), and the Chomsky hierarchy (Lin, 2022). Meanwhile, many other philosophers also addressed the interdisciplinary transfers of models, knowledge, or facts, though not using the notion of a template as their main unit of analysis (Herfeld & Doehne, 2019; Herfeld & Lisciandra, 2019; Howlett & Morgan, 2010; Jhun et al., 2018; Zuchowski, 2019).

While these analyses of model transfer and migration provide a fresh and valuable perspective on contemporary modeling practice, we argue, as anticipated in Humphreys (2002, 2004), that there is a bigger picture yet to be targeted. The focus on model/template transfer thus far has tended to focus on the exchange of particular models or templates between two or more disciplines. Such a starting point attends to the questions concerning the underlying motivation, representational success, and creativity of particular model/template transfers (Houkes & Zwart, 2019). However, the bigger picture we are after here is to gain a better understanding of the contemporary modeling practice in mathematically and computationally oriented fields that is largely template-based, in both the natural and social sciences.

We argue that what appears to be an interdisciplinary model transfer between different domains turns out, from a broader perspective, to be the application of *transdisciplinary model templates* across a multitude of domains (Knuuttila & Loettgers, 2014, 2016b). With transdisciplinarity, we refer to such research strategies that cross disciplinary boundaries aiming for a more unified approach. The notion of a model template better captures such strategies than the various template notions offered by Humphreys, due to the model template containing both a mathematical structure and a generic conceptualization that makes it able to address a particular kind of phenomenon potentially encountered in various domains. We suggest that it is precisely the entwinement of conceptual, mathematical, and computational resources from which model templates derive their transdisciplinary prowess.

In order to articulate further the notion of a model template, we study three powerful model templates: The Sherrington-Kirkpatrick model (Sherrington & Kirkpatrick, 1975), scale-free networks (Barabási & Albert, 1999), and the Kuramoto model (Kuramoto, 1975a). Based on these case studies and earlier literature on templates, we further articulate the notion of a model template, consisting of its mathematical structure, ontology, prototypical properties and behaviors, focal conceptualizations, and the paradigmatic questions it addresses. We also discuss the entanglement of the three model templates, a phenomenon that has not so far been addressed in the discussion of templates. Scale-free networks and the Kuramoto model have been grounded in statistical mechanics; the origin of the Sherrington-Kirkpatrick model, and the topologies and the dynamics of the three models have been connected in subsequent research.

## Computational templates

Whenever you have a sudden increase in usable mathematics, there will be a sudden, concomitant increase in scientific progress in the area affected.

(Humphreys, 2004, p. 55).

Humphreys (2002, 2004) introduced the notion of a computational template for the analysis of computational science, focusing on how computer simulations are related to traditional mathematical modeling, in particular, to solve equations (see also Humphreys, 2019a, p. 114). He pointed out that the deployment of tractable mathematics drives much of the progress in physical and other sciences, a fact that “ought to be uncontroversial, but is not emphasized enough” (Humphreys, 2004, p. 58). The prominence of a relatively small number of computational templates in the quantitatively oriented sciences is mainly due, according to Humphreys, to their computational tractability (p. 68). Moreover, the use and reuse of the same computational templates across different domains and disciplines is epistemically advantageous, since “science would be vastly more difficult if each distinct phenomenon had a different mathematical expression” (ibid.). Examples of computational templates include the Poisson distribution, the wave equation, harmonic oscillators, the Ising model, the Lotka-Volterra equations, and agent-based models. For Humphreys, such templates offer a new unit of analysis that enables envisioning a different kind of organization of quantitively oriented sciences than the conventional disciplinary matrix.

Humphreys (2004, 2019a) contrasts the notion of a computational template with theoretical templates. The latter are general representational devices that occur within an interpreted theory that is about a particular subject matter (Humphreys, 2019a, p. 114). Theoretical templates constrain the substitution of schematic variables according to the intended interpretation of the theory, and they are often part of the fundamental principles of the theories in question (ibid.). Newton’s Second Law, Schrödinger’s equation, and the Lotka-Volterra equations provide, for Humphreys, examples of theoretical templates. However, the Lotka-Volterra equations do not have the same theoretical status as the two former ones, as these equations can already be considered a computational template. Indeed, Humphreys (2019a) mentions another type of theoretical template that, instead of being fundamental to its respective theory like Newton’s Second Law or Schrödinger’s equation, is itself derived from “fundamental principles of the domain and can be explained entirely in terms of the subject matter of that field” (2019a, p. 114). Such theoretical templates are derived from construction assumptions, involving also a correction set that is not necessarily explicitly formulated, giving guidance to the process of de-idealization, or correcting for abstractions, approximations, and other assumptions. Presumably, the Lotka-Volterra model is an example of the latter kind of theoretical template, though its origins are heterogeneous: both Volterra and Lotka creatively employed resources from other fields, such as mechanics, physical chemistry and even demography (Knuuttila & Loettgers, 2017). Humphreys does not explicitly discuss examples of these less fundamental kinds of theoretical templates, therefore it is unclear what templates he had in mind.

To characterize theoretical and computational templates and distinguish them from each other, Humphreys studies Newton’s Second Law. He shows how computational templates can be drawn from theoretical templates by specifying parameter values as well as general features, for instance, various forces in the case of Newton’s Second Law. If one achieves a mathematically tractable equation through such substitutions, with possible uses across different fields, one has arrived at a computational template. Not all theoretical templates, such as Schrödinger’s equation, can be detached from their original theoretical context and turned into computational templates. But some can, and such computational templates can be found at different levels of abstraction. Many computational templates also trace their origin back to formal fields of inquiry, such as the Poisson distribution and scale-free networks.

In his notion of a computational template, Humphreys (2004) combines two things: tractability and cross-disciplinary applicability, the latter boiling down to the detachability of a template from its original theoretical domain. The two features do not necessarily align, though they often do, since what is tractable may be usable in another context. It is tractable mathematics, related to the inability to analytically solve theoretical templates, rather than interdisciplinarity that is central to Humphreys’ treatment of computational science in his book, *Extending Ourselves* (2004). In rendering non-linear equations more tractable, and allowing for de-idealization, computer simulations enlarge the scope of tractable computational templates. The Lotka-Volterra model provides a good example of the expansion of tractable mathematics that computational science has made possible. Due to its non-linearity, it became a fully-fledged computational template only after the introduction of computers and computational methods, which led to its renaissance through the work of May (May, 1974), for example.

Among the targets of Humphreys’ discussion are the semantic approaches that abstract from the linguistic particularities of syntactic representation. Humphreys aims to show that it is precisely the tractability of a syntactic form that matters in “bringing mathematically formulated theories to bear on natural systems” (Humphreys, 2019b, p. 68).

It is important to keep in mind that for Humphreys, computational templates are not yet models but can be turned into such. According to him, computational models consist of six components.^1^ They are based on a computational template, such as differential or other kinds of equations, or other formal apparatuses and methods. The other components are construction assumptions, the correction set, interpretation, the initial justification of the template, and its output representation. The construction assumptions cover many things: ontology, idealizations, abstractions, and constraints. The correction set indicates, in advance, and often only implicitly, how the template could be adjusted to better fit the empirical data. Consequently, Humphreys views computational models as results of elaborate construction.

## Template transfer and template application

While Humphreys originally addressed computational science with his template notions, other philosophers later picked up the notion of a computational template and applied it to interdisciplinary model transfer. Knuuttila and Loettgers applied Humphreys’ discussion of computational templates to study the templates and concepts that Volterra and Lotka transferred from physics and other domains to construct their respective versions of the Lotka-Volterra model (2011, 2017), which itself turned into a successful template. Furthermore, they examined the transfer of the Ising model via the Sherrington-Kirkpatrick model to neural networks and socio-economic systems (Knuuttila & Loettgers, 2014, 2016b). Several other studies of model transfer followed suit, highlighting different aspects of model transfer, and employing template notions. Houkes and Zwart (2019) studied the application of the Lotka-Volterra model to technology transfer, showing how some of these transfers were more conformist, and some more creative in nature. Price (2019) argued, using the quantum theory of atoms in molecules (QTAIM) as an example, that the application of a template to a new domain requires the construction of a “landing zone” to be successful. Bradley and Thébault (Bradley & Thébault, 2019) distinguished between imperialism and migration in model transfer, of which the latter explicitly requires “re-sanctioning” to allow for the application to a new domain. Lin (2022) examined the transfer of the Chomsky hierarchy to computer science and cognitive biology, finding that the spillover of knowledge claims from former applications could be crucial for the further reapplication of a mathematical construct.

Several of these studies address one particular ambiguity in Humpreys’ original template account (e.g., Houkes & Zwart, 2019; Knuuttila & Loettgers, 2014; Lin, 2022). While discussing computational models, Humphreys appears to consider computational templates as merely tractable formal templates to be complemented by other components of the computational model (2004, p. 103, see above). Yet he approaches elsewhere the construction of computational templates in terms of construction assumptions similar to those of computational models, including an assumed ontology covering “mechanisms that operate within and between systems” (2004, pp. 78–79). Clearly, computational templates understood in this latter way do not boil down to formal templates, and consequently, it is difficult to distinguish computational templates from computational models (Houkes & Zwart, 2019). In order to distinguish between these two interpretations of computational templates, we call them thin and thick notions of a computational template. The notion of a model template comes closer to the thick notion of a computational template, though it underlines the conceptual side of templates, to which Humphreys did not pay too much attention until his final work (Humphreys, 2023).

Humphreys in Humphreys (2019a) turned explicitly to template and model transfer, launching two novel template notions, a formal template and a transdomain template, leaving computational templates aside. Humphreys (2019b, 2023) explains, in line with the thin notion of computational templates, that his “focus was originally on computational aspects of representations and so a primarily formal approach to transfer seemed suitable […]” given that “with only formal application conditions, the tractability of a given model is independent of the application” (Humphreys, 2023). However, he acknowledges that in model transfer, one also needs to pay attention to other constraints related to the empirical adequacy of the model.

Humphreys (2019a) uses network models as examples of *formal templates* as they have their origin in formal fields of inquiry. He claims that in their application, all empirical content is contained in the (often complicated) mappings of the formal template to a particular target system (2019a, pp. 116–117). However, not all cases in which a seemingly identical formal template is applied in different domains qualify as instances of template transfer. Humphreys argues that there are cases in which an identical formal template is arrived at by different, independently justified construction assumptions. Volterra’s population biological and Goodwin’s economic construal of the Lotka-Volterra equations serves as an example of such a case for Humphreys, and he also refers to Lotka’s and Volterra’s different construals of Lotka-Volterra equations (see Knuuttila & Loettgers 2011, 2017). Humphreys calls such templates transdomain templates. It is important to note that in such cases, according to him, there is no template transfer, as the templates are arrived at independently of each other despite their similar formal structure. Finally, Humphreys recognizes that there are “stylized formal templates”—or “off-the-shelf models”—that “are opportunistically justified at the system level by analogical reasoning from their previous successful applications to systems that are recognized as similar” (2019a, p. 115). He does not further contemplate these cases, however, arguing instead that construction assumptions of formal and transdomain templates can be made explicit, and empirically verifiable (at least in principle).

In Humphreys’ (2019a) discussion of template transfer, the theoretical and ontological aspects of templates, as well as the tractability considerations, recede into the background. His focus is on formal and empirical aspects of template transfer which is especially clear in his discussion of formal templates. Since Humphreys (2019a) does not consider the cases of independently derived transdomain templates as instances of model transfer, the earlier cross-disciplinary applicability of computational templates is lost, apart from formal templates. As a result, it becomes unclear why scientists would engage in template transfer and application in the first place. The notion of a model template (Knuuttila & Loettgers, 2014, 2020) seeks precisely to address this question.

A model template is “a formal platform for minimal model construction coupled with very general conceptualization without yet any subject-specific interpretation or adjustment (Knuuttila & Loettgers, 2016a, 2016b, p. 382). The notion aims “to capture the intertwinement of a mathematical structure and associated computational tools with theoretical concepts that, taken together, depict a general mechanism that is potentially applicable to any subject or field displaying particular patterns of interaction” (Knuuttila & Loettgers, 2014, p. 295). The point Knuuttila and Loettgers make is that even in the case of templates originating from formal fields, such as scale-free networks, there is a generic ontology and conceptualization that suggests the kinds of systems and problems it is applicable to, and thus motivates and guides template transfer and application.

The shift from formal and (thin) computational templates to model templates as units of analysis takes into account the theoretical and conceptual resources that templates make available and that facilitate their applications to new domains, providing material for further theoretical development. Furthermore, the notion of a model template also addresses the aforementioned worry that several interpreters of Humphreys’ work on templates have voiced concerning the difficulty of distinguishing computational templates from computational models. The broader notion of a model template appears fruitful because it is often indeed difficult to tell apart templates and models—and in fact templates and methods as well, as we will later show in the case of the Sherrington-Kirkpatrick model. One can discern cases, in which the model itself is the primary object of study, and, on the other hand, cases in which a model is applied to some specific system in a particular domain. In the former cases, the model comes closer to a template, yet it still embodies much more conceptual and theoretical content than a mere formal template does.

Consequently, we consider the notion of a model template as a promising alternative to Humphreys’ computational and formal templates in the analysis of cross-disciplinary model application, though being simultaneously in line with his original vision. In what follows, we seek to articulate the notion of a model template further by developing a categorization of the different constituents of model templates by studying three successful and widely applied model templates: the Sherrington-Kirkpatrick model, the scale-free networks model, and the Kuramoto model.^2^ The respective origins of the three templates in physics, applied mathematics, and the study of synchronization and self-organization, display the heterogeneity of the cross-disciplinary trajectories of model templates. However, despite their different origins, the three templates have also come together, becoming entangled with one another.

## Model templates

The Sherrington-Kirkpatrick model was originally introduced in physics to study spin glasses, later finding applications in as disparate fields as statistical physics, computer science, neural networks, and financial markets (Knuuttila & Loettgers, 2016b, 2020). The scale-free networks and the Kuramoto model can be located in the more formal fields of network science and the study of synchronized oscillators, neither of them tied to any particular discipline. Scale-free networks originated in mathematics, in the investigation of random networks (Erdös & Rényi, 2011). The study of networks only entered physics once the scale-free networks were grounded in statistical mechanics and critical phenomena, in an attempt to assign scale-free networks a universal status (Barabasi, 2018). The Kuramoto model addresses the general phenomenon of synchronized oscillation. The interest in such oscillations goes back to the seventeenth century when the Dutch mathematician and physicist Christiaan Huygens observed the synchronization of two pendulum clocks. The omnipresence of synchronized oscillations in nature made them a subject of increasing interest, especially in mathematically oriented domains (Strogatz, 1994). The introduction of the Kuramoto model in the mid-1970s drew many of these different attempts together, making them mathematically tractable (Kuramoto, 1975a, 1975b). Like scale-free networks, the Kuramoto model was linked to statistical mechanics.

### Sherrington-Kirkpatrick (SK) model

The Sherrington-Kirkpatrick (SK) model (Sherrington & Kirkpatrick, 1975) was constructed to study the properties and behavior of spin glasses. The SK model has been a subject of intense mathematical and theoretical research within statistical mechanics for nearly 50 years (Panchenko, 2012). The model was constructed to study spin glasses that are made of particular kinds of magnetic alloys. It models the competing ferromagnetic and antiferromagnetic interactions taking place in said alloys. These interactions lead to exceptional properties and behaviors, which explains the interest physicists have taken in spin glasses. But the SK model did not remain in physics, it became a model template as it was applied to a large number of different phenomena such as, for example, pattern recognition in neural networks (Hopfield, 1982), social networks, and peer effects (Bertrand et al., 2000; Bramoullé et al., 2009).

The SK model has its origin in the Ising model (Ising, 1925), which also has become a model template in itself (Knuuttila & Loettgers, 2014, 2016b, 2017, 2020). In the Ising model, magnetic moments are described as binary variables located on a grid exhibiting only the nearest neighbor interactions. At high temperatures, the magnetic moments are subject to thermal fluctuations, but with a decreasing temperature, they start to align—the material performing a phase transition from antiferromagnetic to ferromagnetic. The phase transition takes place at a critical temperature *T*_*C*_ and is accompanied by a pronounced increase in magnetic susceptibility $\chi ,$(i.e., a measure of how much a material will become magnetized in a magnetic field), and in specific heat *c* (i.e., the amount of heat needed to raise the temperature by one degree Celsius per unit mass).

Given the interactions among the magnetic moments, the behavior of spin glasses can be analyzed as a particular kind of collective phenomena. Collective phenomena are the results of the interaction between constituents of a system, such as ferromagnetism being caused by the interaction between magnetic moments. The notion of collective phenomena aligns ferromagnetic material and spin glasses, enabling them to be modeled using the same mathematical tools. In constructing the SK model, Sherrington and Kirkpatrick made use of the Ising model. They formalized the phenomenon of disorder resulting from competing interactions between magnetic moments in spin glasses in which neighboring magnetic moments try to align a magnetic moment in different directions, but modified it in such a way that it would allow for the exploration of disorder and frustration in spin glasses. In this instance, frustration is understood as resulting from not being able to satisfy the competing interactions (Parisi, 1986). In the mathematical rendering of the SK model, the interactions are modeled as a function of the distance between the magnetic moments ${S}_{i}$ and ${S}_{j},$ which reads as ${J}_{ij}=J({R}_{i}-{R}_{j})$, with ${R}_{i}$ and ${R}_{j}$ as the positions of the magnetic moments on, for example, a two-dimensional grid. The positive values of ${J}_{ij}$ correspond to ferromagnetic, and negative values to antiferromagnetic couplings. The overall energy of the system is described by the following equation:

1 $$E= -\sum_{i,j}{J}_{ij}{S}_{i}{S}_{j}$$

The disorder and competition among magnetic moments result in a highly structured energy landscape, consisting of a large number of local energy minima. As Mézard et al. write: “The picture which has emerged is that the main characteristic of the glassy phase is the existence of a large number (infinite when the number of spins $N\to \infty$) of equilibrium states $\alpha =\mathrm{1,2},\dots \left[\dots \right]$” (Mezard et al., 1987, p. 13). Due to the disorder and frustration, no ground state can be assigned to spin glasses.

For the SK model to function as a model template, the topology of its rugged energy landscape, with its large number of local energy minima, is crucial. As a model template, the SK model can be applied both to various kinds of empirical phenomena as well as used as an optimization method. An example of the first kind of application is the Hopfield model (Hopfield, 1982), which is an artificial neural network modeling auto-associative memory. In this case, the large number of local energy minima allows for the storage of a large number of patterns.

The application of the SK model to neuroscience brought along concepts from statistical mechanics such as phase transitions, order parameters, critical exponents, and symmetry breaking, as well as specific methods like the method of mean field approximation and the replica symmetry breaking that became detached from their original context (Knuuttila & Loettgers, 2014, 2020, see also discussion below, in Sect. 5). On a more general level, both the SK and Hopfield models can be approached as networks having specific topologies and dynamics. The topology of the energy landscape of the SK model is crucial also for its application to optimization. The research on the SK model has led to computational methods that can be applied to many combinatorial optimization problems, such as NP complete problems^3^ like the traveling salesman problem, matching problem, or protein folding. According to Parisi, “it is quite possible that on the long run the applications of these ideas beyond solid state physics will be the most interesting ones.” (Parisi, 1986, p. 2).

**Fig. 1:**
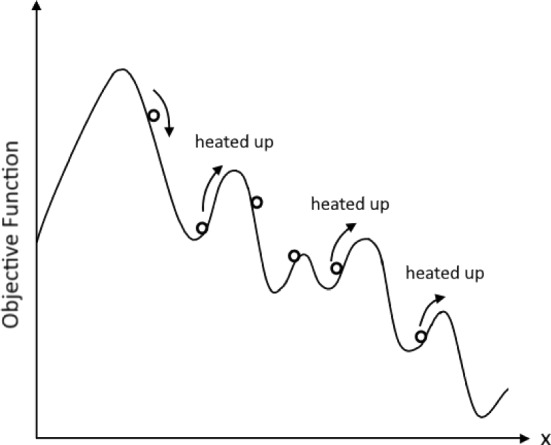
The graph shows a cut through the energy landscape of a system consisting of a large number of local energy minima

The traveling salesman problem consists of calculating the shortest route through different cities, such that the salesman passes every city only once (Fig. 1). It is a combinatorial optimization problem, in which different routes have different lengths and travel costs. In the traveling salesman problem as in the case of the SK model, the goal is to find the true minimum in a landscape of a large number of local minima. What makes the problem belong to the class of NP problems is the fact that the number of possible tours increases rapidly with the number of cities $n$, which is given by $\left(n-1\right)!/2$. In the case of $n=50$ it would take a longer time than that of the existence of our universe to calculate all the different tours.

The method of simulated annealing provides another example of such a computational application. This method is named after a procedure in metallurgy, which aims to find the state of the lowest energy of a metal. To avoid getting trapped in a local energy minimum, the metal is heated up in irregular time intervals during the process of cooling down.

Figure 1 shows a cut through the rugged energy landscape of a spin glass. The objective function in this diagram represents, in the case of spin glasses, the energy function. The local minima of spin glasses can be explored by calculating the probability of a configuration at a specific temperature, or, alternatively in the case of the salesman problem, the probability assigned to a specific route. As shown in the schematic diagram, even when the energy function or the cost function results in a local metastable energy minimum, small perturbations, e.g., heating up in the case of metals, guarantee that the system does not get trapped in them. Larger parts of the energy or cost landscape get explored in this way. Though such a method does not give an exact solution, it can provide a satisfactory approximation (see Hopfield & Tank, 1986).

### Scale-free (SF) networks

According to a common narrative among network scientists, networks provide a natural way of analyzing our highly connected world. This narrative has gained momentum since huge amounts of data have become available from, for example, the human genome project, food webs, the web of human sexual partners, and the spread of infectious diseases. Scale-free networks have become, together with other network models such as small-world (Watts & Strogatz, 1998, p. 440) and random networks (Erdös & Rényi, 2011), central to the field of network science. The differences between these three different network models lie in their respective topologies and dynamics that influence what kind of problems and phenomena they can be applied to.

**Fig. 2:**
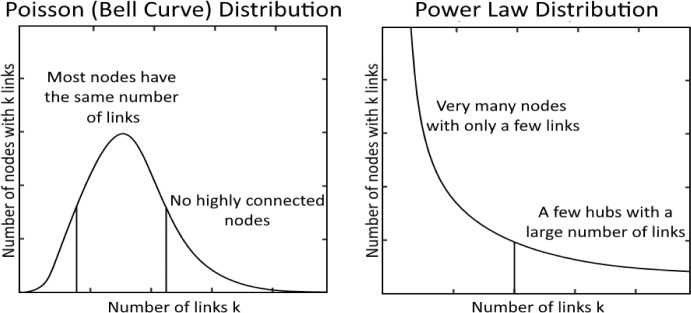
The two curves illustrate the difference between random and power law distributions and their respective effect on the network structure (Glushnev et al., 2003, p. 53)

Crucial to the SF network is the assumption that connections $k$ follow a power law distribution $P(k)\sim {k}^{-\gamma }$, where the scaling parameter $\gamma$ is a constant lying in the range $2<\gamma <3$. In Fig. 2, the peculiar feature of the power law distribution is illustrated in comparison with a random distribution. The left-hand side in this figure depicts a probability distribution for randomly distributed *k* and the right-hand side a probability distribution in which $k$ follows a power law. In the case of the random distribution, the probability distribution of *k* has the form of a bell-shaped curve with the maximum representing the mean value of $k$ and the width representing the standard deviation. This distribution is the well-known normal distribution.

In the second case, the probability distribution follows a power law—the probability exponentially decreases with increasing *k*. A comparison of the two distributions shows that for those values of $k$ where the random distribution is already zero, in the case of the power law distribution still some finite probability exists for *k*$.$ This specific feature is called a fat-tailed distribution. Fat-tailed distributions have the property of decaying very slowly, allowing for more outlier data than is the case with normal distributions. As a result, extreme events such as large earthquakes, blackouts, or stock market crashes are more likely to occur than what would be predicted by normal distributions.

**Fig. 3:**
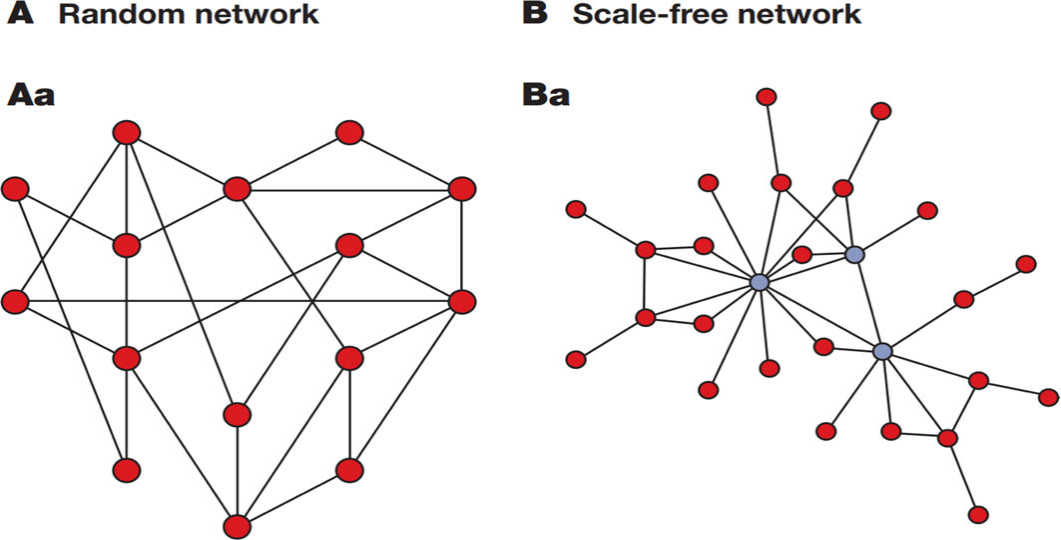
The illustrations show the difference in the distributions of nodes in a random and a scale-free network. Some dots in the scale-free network represent the highly connected hubs (Barabási & Oltvai, 2004)

In the SF network, a power law distribution allows for the formation of a few highly connected hubs. This is not the case in a random network, where the connections between the nodes follow a normal distribution. Figure 3 shows a comparison between a random and a SF network. In the topology of the SF network, some nodes are more connected (i.e., “hubs”) and others are less connected. Because of the high connectivity of hubs, SF networks are highly robust against the random removal of the connections between the nodes*.*

When Barabási and Albert introduced the SF network in 1999 (Barabási & Albert, 1999), there were already available data on protein networks of yeast and the World Wide Web (WWW).^4^ Using a robot to detect which web pages in the WWW are connected to each other, they claimed that they were able to directly infer from the data that the topology of the WWW has a small number of highly connected nodes, the “hubs”. Barabási and Albert claim that for large values of $k$ the degree distribution follows a power law $P\left(k\right)\sim {k}^{-\gamma }$. They extended their empirical findings to cover various kinds of systems, arguing that such unrelated networks as scientific networks, gene regulatory and protein networks, and food webs also show similar topologies that follow a power law and are thus by definition scale-free (Albert & Barabási, 2002).

It is important to note that the connection between power law distribution and scale-freeness is central to statistical mechanics and phase transitions. Indeed, in grounding SF networks in statistical mechanics, Barabási and Albert sought to give an ontic, and even a universal interpretation of networks. Such universality would justify the application of SF networks to various kinds of systems, a tendency that can be observed in network science more generally. To justify such a move, Albert and Barabási invoke the earlier work on random networks, which are supposed to produce second order phase transitions (continuous phase transitions) if sufficiently many links have formed in the network (Albert & Barabási, 2002). The phase transition is supposed to result from an ‘explosive-percolation,’ meaning that a large fraction of nodes suddenly become connected. In the case of scale-free networks, the question then becomes whether they also exhibit phase transitions, and on what parameters such transitions might depend. The link between networks and statistical mechanics made physics concepts such as phase transition, symmetry breaking, order parameters, and critical exponents available for network theorists. As a result, these concepts became independent from their specific meaning in physics—as was also the case with the Hopfield model. In contrast to the Hopfield model, network models such as the SF are more general and abstract and do not address any particular system.

While Albert and Barabási understand SF networks as an extension of models such as the Ising model and SK spin glass model, they nevertheless point out that models with static grids and equidistant nodes do not apply to networks such as the WWW or chemical networks in cells: “The success of these modeling efforts (Ising and SK model) is based on the simplicity of the interactions between the elements: there is no ambiguity as to what interacts with what, and the interactions are uniquely determined by the physical distance. We are at a loss, however, to describe systems in which physical distance is irrelevant or for which there is ambiguity as to whether two components interact” (Albert & Barabási, 2002, p. 2). Instead of the distance of nodes, it is the distribution of the connections between the nodes that is crucial in the SF network.

The main idea of how the nodes become connected in the SF network over time is captured in the saying, *‘the rich are getting richer’*. The probability that a new node will be connected to one of the nodes in the network depends on the number of connections the node has to other nodes. This phenomenon is called preferential attachment. While Albert and Barabási seek to accumulate evidence for the omnipresence of SF networks in real-world systems, this claim has been met with criticism. Scientists do not usually have access to the complete network, and it is questionable whether they can infer from the distribution of the sub-network the distribution of the whole network (Stumpf et al., 2005). Moreover, the identification of power law distributions from real data is difficult. For example, such an identification could be complicated by fluctuations, especially in the tail of the distribution (Clauset et al., 2009). Consequently, it does seem that the universality of scale-free networks should be taken with a grain of salt.

### The Kuramoto model

The Kuramoto model (Kuramoto, 1975a, 1975b) is a model of coupled oscillators developed by Yoshiki Kuramoto to study synchronization between different oscillatory systems. Synchronized oscillations belong to the larger and more general class of such phenomena as synchronized behavior and self-organization. There are many different instances of such synchronization in nature as well as in engineered systems. On an abstract level, synchronization describes a specific relatedness in dynamical systems—they become synchronized in case phenomena occur at the same time. In movies, the flow of pictures and sound is synchronized, birds and fish show swarming behavior, and our different electronic devices such as computers and mobiles are often synchronized. Examples of synchronized *oscillations*, in turn, are the millions of fireflies in Southeast Asia blinking in sync, an audience after a play falling in sync when clapping their hands, or the circadian clock, which is entrained by the environment and regulates the various processes taking place in an organism in a 24-h cycle (Strogatz, 2003).

Synchronized oscillations have been characterized by Pikovsky as the “adjustment of rhythms of oscillating objects due to weak interactions” (Pikovsky et al., 2002). The dynamics of oscillatory systems are examined mathematically by the dynamics of the phases of single and coupled oscillators in phase space (Strogatz, 1994). The following example may be helpful. Each of the oscillators can be thought of as a runner on a circular running track. In the unsynchronized case, the runners orbit the circle with different frequencies (i.e., different speeds) and phases (i.e., different positions on the track). In the synchronized case, the frequencies and phases of the runners coincide. The coupling between the oscillators is modeled as a coupling between their phases. The resulting dynamic of the phases of the oscillators—i.e., the change in the phase of the oscillators over time—is described by a trajectory in the high dimensional phase space, which entails all possible uncoupled, coupled, and synchronized states of oscillators.

The mathematical tractability of the Kuramoto model is far from obvious. Kuramoto was able to solve exactly a system of infinitely many differential equations, all nonlinear and coupled together. The model consists of a system of coupled non-linear differential equations of the following form:

2 $$\frac{d{\theta }_{i}}{dt}={\omega }_{i}+\frac{K}{N}\sum_{j=1}^{N}\mathrm{sin}\left({\theta }_{j}-{\theta }_{i}\right)\quad \quad i=1,\dots ,N$$

In this system, ${\theta }_{i}$ is the phase of oscillator $i$, ${\omega }_{i}$ its frequency, and K, the coupling strength between the oscillators. The non-linearity enters the model by $sin$ function. In the original version, the oscillators are identical and located on a one-dimensional grid (string). The oscillators are coupled by their phases via the $sin$ function.

Kuramoto’s derivation of his model of synchronized oscillators was inspired by Arthur Winfree’s earlier model (Winfree, 1967) that addressed synchronized phenomena, such as synchronization in pacemaker cells and the circadian clock, or blinking fireflies. He suggested conceptualizing the transition from an unsynchronized to a synchronized state as a phase transition (Winfree, 1967, 2001). In a remarkable feat, Kuramoto was able to tie the problem of synchronization to statistical mechanics, making the model computationally tractable in one stroke. As such, Kuramoto’s model provides yet another example of transferring the concepts of phase transition, symmetry breaking, order parameter, and critical exponents into the research of complex networks.

The tractability of the model depends on what is called the order parameter of the system, a value that indicates how synchronized the system is. In statistical mechanics, this value indicates the occurrence of a phase transition. Kuramoto employed the mean field method, in which a system of interacting entities is described as independent entities coupled to a mean field. The technical details do not concern us here, but it is important to recognize that with the order parameter, the phases and, thereby the oscillators, become decoupled and consequently computationally tractable.

Because of its computational tractability and flexibility, the Kuramoto model is a powerful computational template. It can be found in a large spectrum of research areas such as neuroscience, condensed matter physics, cell and molecular biology, sociology, and electrical engineering. Over time, the Kuramoto model has been expanded from a one- to a two-dimensional grid (Wiley et al., 2006), from identical to non-identical oscillators (Brede, 2008), and made to cover different network topologies such as small-world networks (Watts & Strogatz, 1998). Like SF networks, the Kuramoto model has some grounding in statistical physics, getting its model template status from this conceptual framework, in addition to the previous work on synchronized oscillators.

## A categorization of the constituents of model templates

The previous discussion of the three models shows that each model can be considered a model template due to the way their mathematical structure intertwines with computational methods and theoretical concepts. Table 1 provides our categorization of the different constituents of a model template aiming to analyze the notion discussed in the literature so far (Knuuttila & Loettgers, 2014, 2016b). In Table 1, we distinguish between the mathematical structure, ontology, prototypical properties and behaviors of model templates, and their generic and system-specific conceptualizations, as well as the paradigmatic problems the templates are used to address, mentioning also some applications, all of them discussed in our presentation of the cases above. We do not claim this categorization to be exhaustive.

**Table 1:** The constituents of model templates

Dimensions of the model template	Sherrington-Kirkpatrick model	Scale-free networks	The Kuramoto model
Mathematical structure:	The overall energy of the SK model is given by: $E=-{\sum }_{i,j}{J}_{ij}{S}_{i}{S}_{j}$, with ${J}_{ij}=J\left({R}_{i}-{R}_{j}\right),$ depending on the distance between the magnetic moments	Network topology evolves following a preferential attachment mechanism: the connection between the nodes is given by a power law distribution $P(k)\sim {k}^{-\gamma }$	Non-linear coupled differential equations: $\frac{d{\theta }_{i}}{dt}={\omega }_{i}+\frac{K}{N}\sum_{j=1}^{N}\mathrm{sin}({\theta }_{j}-{\theta }_{i})$
Ontology:	Magnetic moments are modeled as binary variables, taking values + 1 or −1. The magnetic moments are placed on a grid and their coupling is modeled as a function of the distance between the magnetic moments ${S}_{i}$ and ${S}_{j,}.$	Network ontology of nodes and edges, whose distribution follows the power law and is thus scale-free	Identical oscillators, which are either placed on a grid or some network, such as small-word networks. The coupling is modeled via the phases of the oscillators
Prototypical properties and behaviors:	A rugged energy landscape consisting of a huge number of metastable states, which are due to disorder and frustration in the interaction between magnetic moments This specific property finds its expression in the topology, in which the magnetic moments are placed on the edges of a grid in random directions	Networks containing hubs, with a high degree of connectivity. These networks are highly robust against the random removal of the connections between the nodes In this case, the rigorous topology of a grid is replaced by a more dynamical topology	A phase transition from unsynchronized to synchronized oscillations. The Kuramoto model was the first model to exhibit such transitions The topology of the model is flexible because different kinds of grids, as well as small-world, or scale-free networks, can be implemented within this framework
Conceptualizations: Generic System-specific	Cooperativity, phase transitions, symmetry breaking, criticality and critical exponents, phase transitions Temperature and its different re-interpretations (e.g., as noise), disorder, and frustration	Network topology and dynamics, phase transitions, symmetry breaking, critical exponents, universality Hubs, scale-freeness, power law	Nonlinear dynamics, phase transitions, attractors Synchronicity among identical and non-identical oscillators
Paradigmatic problems:	Clustering, collective phenomena, and combinatorial optimization problems	Formation of hubs, modeling the evolution of structure, and robustness in complex networks	Interaction leading to synchronization and self-organization
Examples of applications:	Pattern recognition in neural networks, peer group behavior, welfare participation, and school achievements Optimization problems: traveling salesman problem, matching problem, scheduling	Biological, ecological, and social networks such as neural networks, chemical networks in cells, food webs, celebrity and academic networks, and WWW	Power systems analysis, brain dynamics, synchronizing Josephson junctions in superconducting materials, synchronization in chemical oscillators

Mathematical structure forms the core of the model template. Together with ontology, covering basic objects and relations, and the prototypical properties and behaviors of the model template, it leads to a particular topology that is crucial for the application of the template. Typically, such applications address some paradigmatic general problems across different disciplines, such as collective phenomena, clustering, formation of hubs, synchronization, and self-organization. Generic conceptualizations apply independently from a specific research context, typically deriving from a more general theoretical framework, such as statistical mechanics. The system-specific conceptualizations are those that are crucial for identifying the template in question, and may also need considerable reinterpretation in various applications—showing how any subject-specific application of a template typically involves elaborate model construction.

Below we illustrate the categorization of the constituents of model templates, using the SK model as an example. Due to reasons of space, we cannot discuss the other two models here, but do hope that the preceding discussion and Table 1 indicate how they function as model templates. The peculiar topology of the SK model is a key to its twofold function as a template for modeling various empirical phenomena, and a method of solving combinatorial optimization problems. As an example of the former, in constructing his model of auto-associative memory, Hopfield saw an opportunity of making use of the topology of the SK template to model the storage of a large number of patterns. He turned magnetic moments into neurons and rendered them as binary variables, although neurons in biological networks are non-linear units. Neurons can be made binary by introducing a threshold: if the summed-up signals exceed a threshold, the neuron fires an action potential and goes over into a quiet state (see Knuuttila & Loettgers, 2014).

The topology of the SK model enables it to address particular kinds of *paradigmatic problems* such as clustering and optimization. As for *conceptualizations*, the SK model is firmly rooted in statistical mechanics, exemplified by its generic concepts of collective phenomena and phase transitions. The central concept of collective phenomena allows for different kinds of constituents and interactions and so it did not require any reinterpretation in the case of the Hopfield model. The concept of phase transition in the Hopfield model describes the transition from a state in which the network can recognize a stored pattern to a state in which it loses this ability. The critical parameter, in turn, refers to the storage capacity of networks, e.g., the number of patterns that can be stored in the network. On the other hand, *system-specific* concepts like temperature needed more thorough and less straightforward reinterpretation. In neural networks, temperature has been interpreted as noise, hindering pattern recognition. In contrast, in its use as a computational method for combinatorial optimization problems, the conceptual side of the SK model template is not so important. The topological similarity between the rugged energy landscape of the SK model and the landscape of metastable states of the optimization problems is crucial.

## Template entanglement

What we find particularly interesting in our study of the three model templates—the SK model, SF networks, and the Kuramoto model—is that they are not isolated templates. Even though they originated from different domains, they also have become entangled with one another. This entanglement has happened both through establishing connections between the topologies and dynamics of these templates and through the adoption of the conceptual framework of phase transitions from statistical mechanics.

Albert and Barabási sought to link their networks to statistical mechanics by making them scale-free via possessing a specific topology in which the distribution of the nodes follows a power-law distribution. Scale-freeness is a generic concept from statistical mechanics by which systems, whose patterns are independent from scale, become assigned to universality classes in which the universality corresponds to the behavior of systems as they approach a critical point, such as the phase transition in magnetic systems and liquid–gas transitions at their respective critical temperatures. In the case of these two systems, they are described by the same power law sharing a critical exponent. Different universality classes are distinct from each other by the critical exponent assigned to them. Via the property of scale-freeness, the conceptual framework of statistical mechanics was thus introduced to SF networks, although concepts such as symmetry breaking and phase transition remain vague in this context. Moreover, their properties are still subject to intense debate (Broido & Clauset, 2019; Zhang et al., 2015). SF networks are often applied as “plug-in” templates, being applied to big data collected in, for example, high-throughput omics studies, co-authored works, or the World Wide Web.

The topology of the original Kuramoto model agrees at the outset with the topology of Ising-style models. The oscillators are equidistantly located on a grid and interact with each other. The interaction between the oscillators generates collective phenomena, expressed in the Kuramoto model as a phase transition from unsynchronized to synchronized oscillations. This occurrence of a phase transition depends on the coupling constant between the phases of the oscillators. By approaching synchronization as a phase transition, Kuramoto related his model to statistical mechanics. With this move, the Kuramoto model became embedded into the conceptual framework of statistical mechanics, though mainly as a result of making it computable. But the entanglement of the Kuramoto model extends also to network models due to the flexibility of its topology. The constituents, identical or non-identical oscillators, can be placed on a grid, like the magnetic moments in the SK model, or they can become the nodes in a SF or small-world network.

In referring to this entangled set of models, Watts and Strogatz point out that:

[n]etworks of coupled dynamical systems have been used to model biological oscillators, Josephson junction arrays, excitable media, neural networks, spatial games, genetic control networks and many other self-organizing systems. Ordinarily, the connection topology is assumed to be either completely regular or completely random. But many biological, technological and social networks lie somewhere between these two extremes. (Watts & Strogatz, 1998, p. 441)

Watts and Strogatz’s small-world networks inhabit this middle ground. They are arrived at “rewiring” regular networks by introducing increasing amounts of disorder. These developments have had a feedback effect on spin glass research and the SK model as researchers have used small-world or scale-free networks in modeling the properties of such systems. We suggest that the entanglement between model templates during their development as well as in their application appears as an important characteristic of modeling practices making use of templates. Template entanglement is apparent in the development of a novel transdisciplinary research field devoted to the study of dynamical processes in complex networks (e.g., Barrat et al., 2008). Within this field, the properties of existing model templates are explored, and novel model templates are constructed from existing ones and applied to physical, chemical, biological, social, and ecological systems. The entanglement between model templates is an essential part of this transdisciplinary modeling practice in which model templates themselves are subject to changes and extensions. The implications of such entanglement cut deep: what is at stake may come down to a new kind of template-based unification yet to be addressed by philosophers of science.

## Conclusions

The omnipresence of the same basic equations, function forms, algorithms, and quantitative methods is one of the most striking characteristics of the contemporary quantitatively oriented sciences making heavy use of modeling. We have suggested that the notion of a model template provides a suitable unit of analysis for studying the transdisciplinary nature of present modeling practices. By combing theoretical discussions and case studies of three widely applied model templates, the Sherrington-Kirkpatrick model, scale-free networks, and the Kuramoto model, we have articulated the notion of a model template by developing a categorization of the constituents of a model template. This categorization includes the mathematical structure, a generic ontology, the prototypical properties and behaviors, the core conceptualizations of a template as well as the paradigmatic problems to which the template can be applied. Many of these features are immanent in the mathematical structure of model templates, being crucial for their application to different domains.

The notion of a model template covers but does not reduce to what Houkes and Zwart (2019) describe as a “thin” intentional interpretation of a template that they studied in the context of the transfer of the Lotka-Volterra template. The “thin” intentional interpretation leaves open the precise mechanism and entities at work, yet makes use of some generic features of the interaction between the entities, such as mutualism, predation, and competition. Moreover, the model template notion draws together and unifies the different features of Humphreys’ notions of computational and formal templates. As discussed in Sects. 2 and 3, Humphreys used the notion of a computational template to approach the organization of computational science, and the notion of a formal template when discussing model transfer. The model template notion recovers some crucial aspects of Humphreys’ thick notion of a computational template such as ontology understood in terms of the generic mechanism in operation, but goes beyond it in including also focal conceptualizations of a template and the paradigmatic questions it is used to address. In his latest paper, Humphreys (2023) appears to accept the usefulness of the notion of a model template in writing that “in order to properly account for model transfer, the vehicle of transfer must be taken to be model templates rather than formal templates.”

The constituents of the notion of a model template are articulated at such a level of abstraction that would allow conceiving of model templates as genuinely transdisciplinary devices that can be used for model construction to address particular kinds of problems and phenomena across the disciplinary spectrum. While we acknowledge the importance of analogical reasoning in model and template transfer, the advantage of the template-based approach is precisely due to its transdisciplinary scope. Moreover, the notion of a model template also allows the entanglement of transdisciplinary templates to come into view: addressing the entanglement of templates requires a broader perspective on templates than viewing them as merely mathematical and computational objects able to be transferred from one domain to another.

As we have argued, the usefulness and applicability of model templates accrues, apart from their computational benefits, also from the conceptual, theoretical, and computational resources they offer. But what justifies, beyond such resources, the transdisciplinary application of model templates to particular kinds of problems mushrooming across different disciplines? What makes us think that the seemingly similar phenomena are really due to similar kinds of general principles? Many complexity theorists and network scientists tend to suppose that the same basic organizational principles and topologies govern most diverse materially different systems. Such a view motivates, as we have seen, Barabási’s universalist approach to scale-free networks that has nevertheless met a lot of criticism (e.g., Broido & Clauset, 2019). Indeed, different applications of the same model template may vary greatly in terms of their success. While the application of the SK model to auto-associative memory (Hopfield, 1982) has been constitutive for the entire field of neural networks, Hopfield’s further attempt to apply neural network models to earthquakes has not been so fruitful (Herz & Hopfield, 1995). Kuramoto, in turn, did originally consider his model mainly as a mathematical exercise, but the model has since found truly surprising applications, e.g., to the Josephson effect. And the applications have been piling up: from arrays of coupled lasers to power grids and cardiac arrhythmias. Yet, the Kuramoto model cannot be too widely applied given the restrictive conditions that need to be met by any application. What makes it so important is that it provides a well-understood case of spontaneous synchrony. Strogatz (2003) has put this succinctly by pointing out that “[the] Kuramoto model has always been a solution waiting for a problem” (p. 170).^5^ Perhaps this is precisely what can more broadly be said about all model templates: they provide well-understood and properly defined schematic models of some general phenomena and are as such potentially applicable to numerous problems and targets.

## Footnotes

1 Computational models do not need to be based on a computational template, but Humphreys does not discuss this possibility.

2 As our examination of these three models draws on the theoretical discussion and earlier case studies discussed above, the categorization should not be taken as only a result of inductive exercise. Our approach is in line with the philosophical discussion of case study methodology, according to which case studies supply both evidential and interpretative resources for philosophical theorizing (e.g., Kinzel 2015; Knuuttila and Loettgers 2016a).

3 With NP complete problems, Parisi refers to a class of computational problems for which there can be no effective computational algorithm or it has not been found yet.

4 These data only became available by the late 1990s and the early 2000s (Barabási & Albert, 1999).

5 See also Gelfert’s article on Turing patterns as “a model in search of targets” (Gelfert 2018).
